# Association between vitamin D serum levels and adolescent idiopathic scoliosis

**DOI:** 10.1186/1748-7161-9-S1-O45

**Published:** 2014-12-04

**Authors:** Rodrigo Batista, Delio E Martins, Lilian F Hayashi, Marise Lazaretti-Castro, Eduardo B Puertas, Marcelo Wajchenberg

**Affiliations:** 1 Federal University of Sao Paulo, Sao Paulo, Brazil

## Background

Idiopathic scoliosis (IS) is a deformity of the spine that occurs in up to 4% of children during childhood and adolescence. Idiopathic scoliosis is considered multifactorial, and family history may present several individuals affected. We still cannot determine which curves will worsen and at what rate, but some factors, such as age, growth potential and skeletal maturity have been associated with a higher risk of progression. Studies have suggested that a decrease in bone mineral density may be responsible for the appearance and progression of the disease, and some have tried to link vitamin D receptor gene (VDRG) polymorphism to adolescent idiopathic scoliosis (AIS).

## Aim

The goal of this study was to establish an association between serum levels of 25-OH-VitD and the presence of AIS.

## Design

Cross-sectional study.

## Methods

The patients were recruited from the outpatient clinic of our institution during the year of 2013. Children younger than 10 or older than 18, and those carrying neurologic or muscular disorders, congenital deformities or genetic syndromes were excluded. Calcium, phosphorus, creatine, urea and human parathyroid hormone dosage was taken to rule out renal or parathyroid dysfunctions. Patient’s were measured and weighted, and 25-OHVitD levels were determined through electrochemioluminescence by an automated test after an eight-hour fast, during Brazilian spring. The results were compared to a second group composed of healthy individuals.

## Results

The majority of the patients in both groups are non-black females. On group 1 (controls), 63,3% showed abnormal vitamin D levels, while 91% of AIS patients presented low vitamin D level. The mean BMI were 19,6 kg/m2 for controls and 20,3 kg/m2 on group 2. Statistical analysis through unpaired t tests found relevant difference (p<0.0001) between vitamin D levels. The mean and bottom levels of vitamin D were respectively 27 and 13,6 ng/ml for group 1 and 18,8 and 3,13 ng/ml for AIS.

## Conclusions

There are many factors interacting with vitamin D levels and based on our findings an association of serum 25-OHVitD and AIS could be demonstrated.

